# Mitochondrial Dysfunction and Oxidative Stress in Alzheimer’s Disease

**DOI:** 10.3389/fnagi.2021.617588

**Published:** 2021-02-18

**Authors:** Afzal Misrani, Sidra Tabassum, Li Yang

**Affiliations:** School of Life Sciences, Guangzhou University, Guangzhou, China

**Keywords:** mitochondria, oxidative stress, fission, fusion, mitophagy, Alzheimer’s disease

## Abstract

Mitochondria play a pivotal role in bioenergetics and respiratory functions, which are essential for the numerous biochemical processes underpinning cell viability. Mitochondrial morphology changes rapidly in response to external insults and changes in metabolic status via fission and fusion processes (so-called mitochondrial dynamics) that maintain mitochondrial quality and homeostasis. Damaged mitochondria are removed by a process known as mitophagy, which involves their degradation by a specific autophagosomal pathway. Over the last few years, remarkable efforts have been made to investigate the impact on the pathogenesis of Alzheimer’s disease (AD) of various forms of mitochondrial dysfunction, such as excessive reactive oxygen species (ROS) production, mitochondrial Ca^2+^ dyshomeostasis, loss of ATP, and defects in mitochondrial dynamics and transport, and mitophagy. Recent research suggests that restoration of mitochondrial function by physical exercise, an antioxidant diet, or therapeutic approaches can delay the onset and slow the progression of AD. In this review, we focus on recent progress that highlights the crucial role of alterations in mitochondrial function and oxidative stress in the pathogenesis of AD, emphasizing a framework of existing and potential therapeutic approaches.

## Introduction

Alzheimer’s disease (AD) is a neurodegenerative disease that affects millions of people worldwide (Alzheimer’s Association, 2020), for which only symptomatic treatments are currently available. Current estimates indicate that, in the United States, around 5.8 million patients of age 65 years and older are living with AD in 2020 (Alzheimer’s Association, 2020). AD is characterized by the deposition of extracellular senile plaques, neurofibrillary tangles (NFTs), and neurodegeneration, leading to memory impairment and dementia. The exact mechanisms underlying AD remain unclear despite comprehensive attempts to understand its pathophysiology.

The most prominent theory postulates that, in AD, tau and Aβ negatively affect neuronal cells by compromising energy supply and the antioxidant response, causing mitochondrial and synaptic dysfunction. Neuronal activity is highly energy-dependent, and neurons are particularly sensitive to disruption in mitochondrial function (Kann and Kovacs, 2007; Cunnane et al., 2020). In addition, mitochondria produce cellular energy (adenosine triphosphate; ATP) and are also involved in many processes that are important for the life and death of the cell, including the control of second messenger levels, such as calcium ions (Ca^2+^) and reactive oxygen species (ROS) (Roger et al., 2017; Giorgi et al., 2018). Importantly, mitochondrial dysfunction contributes to reduced ATP production, Ca^2+^ dyshomeostasis, and ROS generation. Alterations in mitochondrial dynamics and mitophagy occur in early-stage AD, but the underlying mechanisms are poorly understood. Thus, studies elucidating the mechanisms of mitochondrial abnormalities in AD will facilitate a greater understanding of the pathogenesis of this neurodegenerative disease and potentially contribute to the advancement of therapeutic strategies to protect synaptic activity and subsequent cognitive function. Here, we review studies that suggest a role of mitochondrial dysfunction and the consequent ROS production in AD pathology and provide a context to explain current and future therapeutic approaches. We suggest that improving mitochondrial function should be considered an important therapeutic intervention against AD.

## Oxidative Stress and Mitochondrial Defects in AD

Oxidative stress is caused by an imbalance between the production and accumulation of ROS, which are inevitable by-products of metabolism that represent a double-edged sword in biological systems (Ferrer et al., 2010; Sies and Jones, 2020); under carefully regulated conditions, they can serve essential roles as, for example, signaling agents, but can also damage cells when produced in excessive amounts since they are capable of oxidizing all major biomolecules, including nucleic acids (DNA and RNA), proteins and lipids (Butterfield et al., 2010; Arimon et al., 2015). ROS are defined as chemically reactive oxygen free radicals as well as no radical derivatives of oxygen. Either enhanced ROS production or an impaired antioxidant system can tip the redox balance of the cell toward an oxidative state.

The brain is especially susceptible to oxidative damage due to its high rate of oxygen consumption, elevated levels of polyunsaturated fatty acids (which are easily targeted by free radicals), and relatively high levels of redox transition metal ions; besides, the brain has very low antioxidants levels (Butterfield et al., 2001; Llanos-Gonzalez et al., 2019; Cassidy et al., 2020). ROS has been proven to account for cellular injury in aging and neurodegenerative disorders (Valko et al., 2007). Indeed, the accumulation of Aβ protein induced by ROS in AD causes lysosome membrane degradation and eventually contributes to neuronal death (Zhang et al., 2009). A deficiency of cytochrome c oxidase is the most common defect in the mitochondrial electron transport chain (ETC) in AD, leading to an increase in ROS production, a decrease in energy stores, and a disruption of energy metabolism (Rak et al., 2016). Furthermore, ROS causes inhibition of phosphatase 2A (PP2A) (Elgenaidi and Spiers, 2019), which facilitates glycogen synthase kinase (GSK) 3β activation (one of the kinases involved in tau phosphorylation). Hence increased GSK3β activation might cause hyperphosphorylation of tau and neurofibrillary lesions in AD (Toral-Rios et al., 2020).

Oxidized biomolecule products produced by ROS are far more stable and widely used as ROS markers. Besides, ROS may also be indirectly tested by measuring antioxidant levels or the activity of antioxidant enzymes. In fact, oxidative imbalance and a substantial increase in its by-products have been repeatedly reported in AD. A large body of research has demonstrated that lipid peroxidation, the process in which ROS attacks lipids to produce lipid peroxidation products via a free radical chain reaction mechanism, is greatly enhanced in AD (Pratico et al., 2001; Galbusera et al., 2004). The most extensive lipid peroxidation products studied in AD are reactive aldehydes, including 4-hydroxynonal, malondialdehyde (MDA), and 2-propenal (acrolein), and chemically and metabolically stable isoprostanoids including F2-isoprostanes and F4-neuroprostanes. A substantial increase in MDA was reported in the hippocampus, pyriform cortex (Lovell et al., 1995), and erythrocytes of AD patients (Bermejo et al., 1997; Dei et al., 2002). Measuring MDA levels, which is both easy and cheap to perform, might be of great importance in monitoring AD progression and treatments. Conversely, the markers of oxidative stress that are commonly used in biological samples include protein carbonyls and 3-nitrotyrosine (3-NT) for protein oxidation; thiobarbituric acid-reactive substances (TBARS), free fatty acid release, iso- and neuroprostane formation, 2-propen-1-al (acrolein), and 4-hydroxy-2-*trans*-nonenal (HNE) for lipid peroxidation; advanced glycation end products for carbohydrates; 8-OH-2’-deoxyguanosine, 8-OH-guanosine and other oxidized bases, and altered DNA repair mechanisms for DNA and RNA oxidation. Increased levels of toxic carbonyls, 3-NT, and HNE are among the earliest alterations seen after an oxidative insult in AD (Butterfield et al., 2007b, 2011; Gamba et al., 2019). Several lines of evidence indicate that oxidative and nitrosative stress can lead to changes in vital cellular elements such as nucleic acids, lipids, and proteins. ROS comprise of both radical and non-radical oxygen species produced by a partial reduction of the oxygen, such as superoxide radical anion (O_2_), hydrogen peroxide (H_2_O_2_), hydroxyl radical (HO), nitric oxide (NO), and peroxynitrite (ONOO-). A key source of free radicals is the mitochondrial-resident oxidative phosphorylation, in which electron leakage from the mitochondrial ETC triggers the production of O_2_ (Ray et al., 2012). In addition to these common markers of protein modifications, protein oxidation/nitrosylation can also result in *S*-nitrosylation and methionine oxidation (sulfoxidation). *S*-nitrosylation is the production of the reaction between cysteine moiety and N^2^O^3^ to form an *S*-nitrosothiol (SNO) (Broniowska and Hogg, 2012). The latter is important in redox-based intracellular signaling, and altered SNO-profile has been documented in AD (Zahid et al., 2014).

**FIGURE 1:**
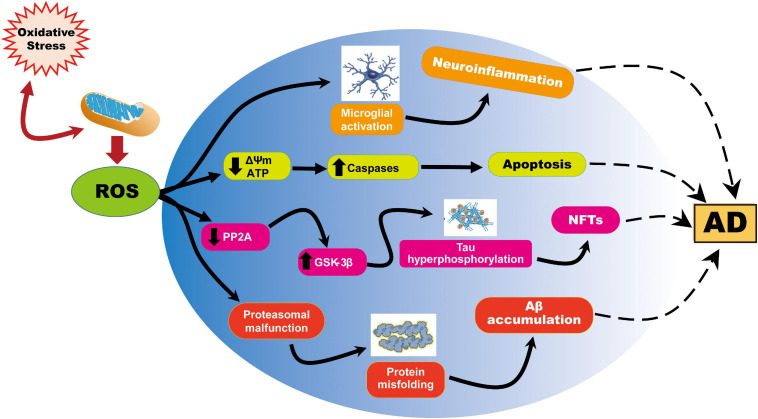
Representation of ROS-induced mitochondrial abnormalities in AD. ROS production or impaired antioxidant system results in the cellular redox balance to oxidative imbalance and cause ROS overproduction. ROS generated during cellular respiration has detrimental effects on mitochondria and neuronal function. Increased ROS causes reduction of mitochondrial ΔΨm and ATP generation through negatively affecting mitochondrial energy stores, disturbance in energy metabolism, and compromised dynamics and mitophagy. ROS further causes an increase in caspase activity and initiates apoptosis. On the other hand, ROS overproduction causes inhibition of phosphatase 2A (PP2A), which also activates glycogen synthase kinase (GSK) 3β causing tau hyperphosphorylation and neurofibrillary tangles accumulation.

In aging and AD, progressive impairment of mitochondrial function has also been implicated as the primary cause of ROS generation; mitochondria are themselves also a major target of oxidative damage (Swerdlow, 2011). In view of the above phenomenon, numerous studies have documented mitochondrial dysfunction through the abnormal processing of ROS as an essential factor in AD pathogenesis (Ohta and Ohsawa, 2006; Selfridge et al., 2013; Wang et al., 2014; Tobore, 2019). Similarly, the insertion of Aβ as oligomers into the bilayer can lead to the development of ROS, thereby initiating lipid peroxidation of membranes, followed by intracellular protein and nucleic acid oxidation (Butterfield, 2002; Butterfield et al., 2007a, Cheignon et al., 2018). It is also important to note that oxidative stress is linked to mitochondrial function, not just because mitochondria generate ROS, but also because ROS can cause deterioration of mitochondrial function (Figure 1). For this reason, reducing ROS levels, using strategies such as diet, exercise, and antioxidant drugs, may protect neuronal mitochondria from oxidative damage and thus reduce the risk of AD.

### Shortage of Neuronal ATP in AD

Mitochondrial ATP production by oxidative phosphorylation (OXPHOS) is essential for cellular functions, such that mitochondria are known as the powerhouses of the cell (Verschueren et al., 2019). The mitochondrial ETC consists of five enzyme complexes in the inner membrane of the mitochondria. ETC generates a charge across the inner mitochondrial membrane, which drives ATP synthase (complex V) to synthesize ATP from ADP and inorganic phosphate.

Several studies have shown impairments of all five complexes in multiple areas of the AD brain (Kim et al., 2000, 2001; Liang et al., 2008). Mitochondrial dysfunction in AD is apparent from a decrease in neuronal ATP levels, which is associated with the overproduction of ROS, and indicates that mitochondria may fail to maintain cellular energy. A substantial amount of ATP is consumed in the brain due to the high energy requirements of neurons and glia. Since an energy reserve (such as fat or glucose) is not available in the central nervous system (CNS), brain cells must continuously generate ATP to sustain neuronal function (Khatri and Man, 2013). Mitochondria are the primary source of cellular energy production, but aged or damaged mitochondria produce excess free radicals, which can reduce the supply of ATP and contribute to energy loss and mitochondrial dysfunction in AD. Importantly, oxidative damage of the promoter of the gene encoding subunit of the mitochondrial ATP synthase results in reduced levels of the corresponding protein, leading to decreased ATP production, nuclear DNA damage to susceptible genes, and loss of function (Lu et al., 2004; Reed et al., 2008).

In advanced stages of AD, substantial nitration of ATP synthase subunits can take place, leading to the irregular function of the respiratory chain (Castegna et al., 2003; Sultana et al., 2006; Reed et al., 2009). Likewise, ATP-synthase lipoxidation occurs in the hippocampus and parietal cortex of patients with mild cognitive impairment (Reed et al., 2008). Compromised OXPHOS contributes to a characteristic mitochondrial dysfunction in AD brains, leading to decreased ATP production, elevated oxidative stress, and ultimately cell death (Reddy, 2006; Reddy and Beal, 2008; Du et al., 2012). The specific mechanisms of OXPHOS deficiency in AD remain a long-standing scientific question, but the role of mitochondrial F_1_F_*o*_ ATP synthase dysfunction in AD-related mitochondrial OXPHOS failure is emphasized by emerging evidence (Beck et al., 2016; Gauba et al., 2019). Therefore, it is important to note that ATP-synthase deregulation caused by ROS is a hallmark of mitochondrial dysfunction in AD, and the strategies to block ROS should prove beneficial by restoring ATP synthase activity.

### Mitochondrial Ca^2+^ Disturbance in AD

Mitochondria play a key role in cellular Ca^2+^ homeostasis, and Ca^2+^ is an important regulator of vital neuronal processes, such as secretion, motility, metabolic regulation, synaptic plasticity, proliferation, gene expression, and apoptosis. The hypothesis that dysregulation of Ca^2+^ homeostasis is a critical factor in accelerating AD pathology is well known. Mitochondrial Ca^2+^ can certainly become a death factor via induction of the permeability transition (PT). The PT is an increase in the permeability of the inner mitochondrial membrane (IMM) to ions and solutes mediated by the PT pore (mPTP), a high-conductance, a voltage-dependent channel that needs a permissive Ca^2+^ matrix load for opening. Persistent opening of mPTP is accompanied by depolarization of Ca^2+^ release, cessation of OXPHOS, matrix swelling with IMM remodeling, and eventually, rupture of the outer mitochondrial membrane (OMM) with the release of cytochrome c and other apoptogenic proteins (Zago et al., 2000; Bernardi et al., 2006; Vercesi et al., 2006; Assaly et al., 2012; Boyman et al., 2019; Carraro and Bernardi, 2020; Nesci, 2020). Understanding the process by which Ca^2+^ is converted from a physiological signal into a pathological effector is one of the outstanding concerns in AD pathology.

**FIGURE 2:**
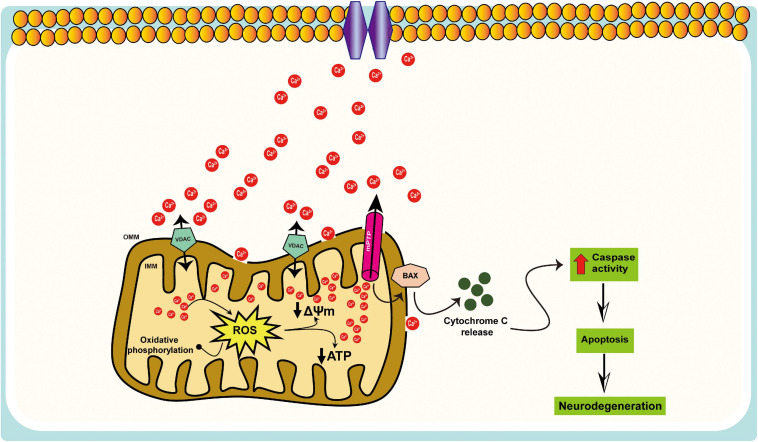
Schematic representation of the mitochondrial Ca^2+^ dysregulation in AD. Mitochondria participate in intracellular Ca^2+^ signaling as modulators, buffers, and sensors; excessive Ca^2+^ taken up by mitochondria can lead to cell death, i.e., mitochondrial Ca^2+^ overload, results in increased ROS production, ATP synthesis inhibition, mitochondrial permeability transition pore (mPTP) opening, the release of cytochrome c, activation of caspases, and apoptosis.

Mitochondria contribute to intracellular Ca^2+^ signaling as modulators, buffers, and sensors (Rizzuto et al., 2012). After a cytosolic rise in [Ca^2+^], mitochondria quickly take up Ca^2+^ to avoid an overload of Ca^2+^ in the cytosol. When excessive Ca^2+^ is absorbed into mitochondria, such that they become overloaded, there is a consequent increase in the production of ROS, inhibition of ATP synthesis, the opening of the mPTP, release of cytochrome c, and activation of caspases and apoptosis (Figure 2). A recent study indicates that Aβ accumulation induces *in vivo* mitochondrial Ca^2+^ overload via the mitochondrial Ca^2+^ uniporter (MCU) complex, leading to neuronal death, and suggests that MCU complex inhibition and blocking the activation of mPTP might represent novel therapeutic approaches toward AD (Calvo-Rodriguez et al., 2020). The increase in mitochondrial Ca^2+^ concentration could contribute to neurotoxicity, but monitoring mitochondrial Ca^2+^ in individual neurons is challenging. Furthermore, mitochondrial Ca^2+^ overload and the resulting dysfunction are a critical cause of apoptosis following ischemic and traumatic brain injury (Rao et al., 2015; Nichols et al., 2018; Novorolsky et al., 2020), as well as in multiple neurodegenerative diseases, including AD, Parkinson’s disease (PD), Huntington’s disease (HD), and amyotrophic lateral sclerosis (ALS) (Gibson et al., 2010; Pchitskaya et al., 2018; Cortes et al., 2020).

On the one hand, intracellular Ca^2+^ regulates multiple neuronal functions; its dyshomeostasis can trigger neuronal injury and death. Indeed, mitochondrial Ca^2+^ overload and subsequent dysfunction are possibly the most significant injury processes caused by excessive concentrations of cytosolic Ca^2+^. The fundamental role of mitochondrial dysfunction in AD, however, is clear, if many of the pathways underlying it are not. Thus, a significant aim of future studies should be to develop a clearer understanding of how, in the first place, mitochondria come to be at risk, and how this risk can be minimized. Much work needs to be done to form a better picture of the involvement of mitochondrial Ca^2+^ dysregulation in AD pathogenesis.

### Impaired Mitochondrial Dynamics and Mitophagy in AD

**FIGURE 3:**
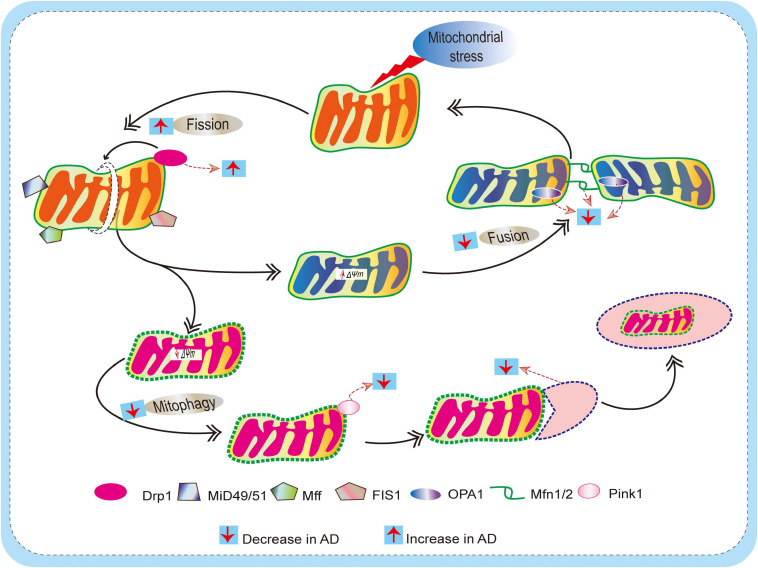
Schematic diagram depicting defects of mitochondrial dynamics (fission and fusion) and mitophagy mechanism in AD. Mitochondria are dynamic, and they undergo frequent changes in shape, size, number, and location to maintain mitochondrial biology and quality control. Actions of outer membrane Drp1 control mitochondrial fission. Drp1 is recruited by mitochondrial fission factor (Mff), mitochondrial fission 1 protein (Fis1), mitochondrial dynamics protein 49/51 (MiD49/51) to promote the mitochondrial fission process. On the other hand, Mitochondrial fusion is regulated by mitofusin (Mfn) 1 and 2 and optical atrophy protein 1 (OPA1). This allows for the exchange of material (matrix components and damaged mitochondrial DNA), as well as promoting a balance in bioenergetic properties (e.g., mitochondrial membrane potential ΔΨm). These fission/fusion processes also involve mitophagy mechanisms (removal of damaged mitochondria) to maintain quality control. When mitochondria become damaged due to cellular stress, sustained depolarization of their inner membrane occurs, resulting in the loss of ΔΨm, which stabilizes PINK1 at the outer membrane to initiate mitophagy. In AD, due to excessive ROS burden on neurons, impaired fission/fusion balance occurs, resulting in defective mitophagy.

Mitochondria are dynamic, and undergo frequent changes in shape, size, number, and location. The various shapes result from the ability of mitochondria to divide, join together, and move throughout the cytoplasm. These processes are collectively referred to as mitochondrial dynamics and largely comprise two unique, closely controlled adverse processes, i.e., fission (division) and fusion (Youle and van Der Bliek, 2012; Palikaras et al., 2018; Chu, 2019; Reddy and Oliver, 2019), both of which are fundamental aspects of mitochondrial biology and quality control (Detmer and Chan, 2007; Youle and van Der Bliek, 2012; Fu et al., 2019). The equilibrium between fission and fusion is important not only for mitochondrial morphology, but also for the viability of cell and synaptic activity. Perturbations in mitochondrial fission, fusion, motility, and turnover can lead to defects in neurons. Previous studies indicate that mitochondrial fusion is neuron protective, leading to the exchange of mitochondrial DNA, reorganization of mitochondrial cristae, and protect cells from apoptosis, whereas mitochondrial fission seems a sign of apoptosis and fragmentation. It is important to note that the fission/fusion process is closely related to mitochondrial mobility and positioning. Likewise, abnormalities in mitochondrial fission and fusion, and consequent changes in mitochondrial morphology, influence mitochondrial mobility and distribution (Chen and Chan, 2009). Additionally, fission and fusion modulate mitochondrial shape, membrane topology, and intramitochondrial protein distribution, which further influence the apoptotic permeability of the mitochondrial OMM (Weaver et al., 2014; Renault et al., 2015). Disruption of fission and/or fusion processes is found in various neurodegenerative disorders including AD, PD and HD (Itoh et al., 2013; Van Laar and Berman, 2013; Hroudova et al., 2014; Reddy, 2014; Bertholet et al., 2016; Stanga et al., 2020). In this section, we discuss in detail the defects in fission/fusion and mitophagy in AD (Figure 3).

#### Mitochondrial Fission Defects in AD

Mitochondrial fission mainly involves the action of GTPase-related dynamin-related protein 1 (Drp1), which is conserved from yeast to mammals (Bleazard et al., 1999; Smirnova et al., 2001). Drp1 is recruited to the OMM by resident protein receptors, including mitochondrial fission factor (Mff), mitochondrial fission 1 protein (Fis1), and mitochondrial dynamics protein 49/51 (MiD49/51) to promote mitochondrial fission (Loson et al., 2013). In general, Drp1 is important in neurons for mitochondrial division, size, shape, and distribution, from the cell body to the axons, dendrites, and nerve terminals. In addition, it is proposed that Drp1 protein is crucial in preserving equilibrium in mitochondrial dynamics, managing fission, mitophagy, and even motility. It mainly acts as a mitochondrial fission factor, triggering mitochondrial fragmentation, and thus when it is downregulated, fusion is partially facilitated (Wenger et al., 2013). Mitochondrial dysfunction that can be attributed to an imbalance in Drp1 activity characterizes the pathology of various disorders including AD, Down syndrome (DS), multiple sclerosis (MS), ALS, PD, and triple-repeat diseases such as HD, spinobulbar muscular atrophy (SMA), spinocerebellar atrophy 1 (SCA1), and others (Wang et al., 2008, 2009b; Manczak et al., 2011; Reddy et al., 2011; Hu et al., 2017). Similarly, elevated Drp1 levels are seen in disease states that cause excessive mitochondria fragmentation, leading to mitochondrial dysfunction and neuronal damage. More specifically, prolonged mitochondrial fission and its deleterious effects are clearly illustrated in *in vitro* AD models: overexpression of amyloid precursor protein (APP) or Aβ treatment causes profound fragmentation and altered distribution of mitochondria, which likely trigger Aβ-induced synaptic defects in neuronal cultures (Wang et al., 2008, 2009a; Manczak et al., 2011). Likewise, experiments in Drosophila have reported Aβ-induced defects in mitochondrial dynamics and distribution as early events *in vivo* (Iijima-Ando et al., 2009; Zhao et al., 2010). Indeed, enhanced mitochondrial fission is well documented in AD patients and model organisms with a bias toward increased mitochondrial fragmentation (Calkins et al., 2011; Manczak et al., 2011; Kandimalla et al., 2016; Manczak et al., 2016). Similarly, increased mitochondrial ROS production, compromised mitochondrial function, and apoptosis has been associated with excessive mitochondrial fission and mitochondrial structural abnormalities (Pena-Blanco et al., 2020). Given the critical role of mitochondrial dynamics, any defects in the fidelity of the fission machinery may have a devastating impact on redox homeostasis, energy generation, and mitochondrial function.

#### Mitochondrial Fusion Defects in AD

In mammals, the fusion of the OMM requires the action of dynamin-related GTPase proteins mitofusin-1 (Mfn1) and mitofusin-2 (Mfn2), which bind the outer membranes of two mitochondria, while inner membrane fusion is facilitated by the optic atrophy type 1 (OPA1) protein. It has been shown that the heptad repeat region of Mfn1 contains an antiparallel coil that is likely to be involved in tethering mitochondria throughout the fusion process (Koshiba et al., 2004). Apart from tethering mitochondrial membranes, Mfn2 serves additional cellular functions, such as mitochondrion-ER contact site development and stabilization, mitochondrion-lipid droplet interaction, cell proliferation, metabolic signaling, and mitophagy (Chen et al., 2014; Zorzano et al., 2015; Naon et al., 2016; Boutant et al., 2017; McLelland et al., 2018). Maintenance of the membrane potential (ΔΨm) of the IMM is necessary for mitochondrial fusion. The mechanistic relationship between ΔΨm and fusion remains to be resolved, but one consideration seems to be the dependency on the ΔΨm of post-translational OPA1 processing (Ishihara et al., 2006). In addition to its function in membrane fusion, OPA1 is also essential for preserving the organization and structure of the IMM (Frezza et al., 2006). Defects in Mfn1, Mfn2, and OPA1 have been reported in various neurodegenerative disorders, including AD. Thus, modulating the activity of these fusion proteins might have an effect on the interaction of the mitochondrial network and IMM structure, both of which affect mitochondrial functions critical for cell health and viability, such as OXPHOS and permeabilization of the membrane during apoptotic cell death.

Recent studies suggest that there is an intrinsic link between hyperphosphorylated Tau and mitochondrial alterations. Mitochondrial dysfunction is detected in P301L tau transgenic mice (David et al., 2005). Abnormal mitochondrial fusion was associated with the overexpression of Tau protein (Li et al., 2016; Kandimalla et al., 2018). Tau ablation resulted in decreased ROS, increased fusion but decreased fission, inhibited mPTP and cyclophilin D (Cyp-D), and enhanced ATP production in mice. The fact that tau indirectly resulted in elongated mitochondria (citation) could be due to tau binding and stabilizing the actin cytoskeletal, disrupting the physical association of mitochondria and Drp1, thus preventing Drp1 dependant fission. The mechanism may involve decreased fission and increased fusion, suggesting that fusion beyond its physiological limit may be detrimental to mitochondrial function. Therefore, preventing tau modifications could enhance mitochondrial health and reduce neurodegeneration.

#### Impaired Mitochondrial Biogenesis in AD

Mitochondrial biogenesis is the process by which mitochondria increase in number and size. A constant renewal of mitochondria is central to maintaining the number of healthy mitochondria. One of the important mitochondrial biogenesis factors is peroxisome proliferator-activated receptor gamma coactivator 1 (PGC-1), a transcriptional coactivator that controls specific transcription action factors, sequentially, coordinating the expression of key nuclear-encoded mitochondrial genes that are required for the proper functioning of the organelle. PGC-1α and estrogen-related receptor-α (ERRα) together stimulate the function of Mfn2 to facilitate the fusion process (Soriano et al., 2006). Repression of PGC-1α and Mfn2 causes a decrease in oxygen consumption, glucose oxidation, and ΔΨm, and an increase in the expression of oxidative phosphorylation proteins (Chen et al., 2005). Moreover, PGC-1α activity is responsive to multiple stimuli, including but not limited to nutrient availability, Ca^2+^, ROS, insulin, estrogen hormone, hypoxia, ATP demand, and cytokines (Huang et al., 2020). Besides PGC-1α, other members of the PGC-1 family of coactivators, namely PGC-1β and PGC-related coactivator (PRC), are also implicated in modulating mitochondrial function, but their exact role is not well understood (Scarpulla, 2008). The capacity of mitochondrial biogenesis declines with aging and in neurodegenerative disease. Notably, decreased levels of PGC-1α and Mfn2 have been reported in AD (Qin et al., 2009; Wang et al., 2009a). The activity of PGC-1α can be modulated by posttranslational signalings such as AMP-activated kinase (AMPK), Akt, p38 MAPK, and the sirtuin 1 (Sirt1). Direct phosphorylation by AMPK activates PGC-1α and promotes PGC-1α dependent induction at the PGC-1α promoter (Canto and Auwerx, 2009). Moreover, activation of Sirt1 through caloric restriction induces PGC-1α activity and enhances mitochondrial function (Planavila et al., 2011; Tang, 2016). Importantly, impaired AMPK, Sirt1, and PGC-1α signaling have been implicated in AD pathology, drugs that activate this signaling would provide hope in alleviating AD.

#### Abnormal Mitochondrial Transport in AD

The proper distribution of mitochondria throughout the cell is achieved by the mitochondrial transport mechanism. Mitochondrial transport relies on proteins that exist in the membranes of mitochondria and transport molecules and other factors such as ions into or out of the organelles (Hansen and Herrmann, 2019; Ruprecht et al., 2019). Mitochondrial transport mainly depends on the actin cytoskeleton in budding yeast (Fehrenbacher et al., 2004) and on both actin and microtubules in mammalian cells (Morris and Hollenbeck, 1995; Ligon and Steward, 2000). These transport mechanisms can ensure the proper inheritance and recruitment of mitochondria. Neuronal mitochondrial transport is essential for providing ATP to the sites of synapses and promoting axonal growth, as well asCa^2+^ buffering, mitochondrial repair, and degradation (Lin and Sheng, 2015). Studies with the membrane-potential indicator dye JC-1 indicate that mitochondria with high ΔΨm favorably travel to the anterograde direction, whereas mitochondria with low ΔΨm move in the retrograde direction (Miller and Sheetz, 2004). These migration patterns suggest that active mitochondria are recruited to distal regions with high energy requirements, whereas impaired mitochondria are returned to the cell soma, perhaps for destruction or repair. Multiple kinesin family members and cytoplasmic dynein have been implicated in anterograde and retrograde mitochondrial transport, respectively (Hollenbeck and Saxton, 2005). Moreover, axonal anterograde transport of mitochondria require actions of Mfn2 (fusion protein), and Milton/Miro complex (members of the molecular complex that links mitochondria to kinesin motors) (Stowers et al., 2002; Guo et al., 2005; Misko et al., 2010). In neurons, cellular signaling cues, such as Ca^2+^, ROS, oxygen level, nutrients, and ATP, act to regulate these Milton/Miro proteins and determine mitochondrial movement and position. Although Milton/Miro proteins have been identified as mammalian adaptors responsible for transporting mitochondria by kinesin, additional motor and adaptor proteins also participate in axonal trafficking of mitochondria transport, ensuring proper mitochondrial distribution in the cell (Melkov and Abdu, 2018). Impaired mitochondrial axonal transport contributes to several human neurodegenerative conditions, including spastic paraplegia, Charcot–Marie–Tooth, ALS, HD, PD, and AD (Charrin et al., 2005; Goldstein, 2012; Lamberts and Brundin, 2017; Flannery and Trushina, 2019). In AD, impairment of mitochondrial axonal transport precedes the accumulation of toxic protein aggregates which is linked to disturbed axonal integrity and synaptic function (Stokin et al., 2005; Calkins et al., 2011). While the precise molecular mechanisms underlying abnormal mitochondrial transport in AD remain elucidated, a disturbance in mitochondrial motility is tightly linked with an unbalanced fission/fusion mechanism, increased levels of both Aβ and pTau, and ROS.

#### Mitophagy Defects in AD

Mitophagy, a selective type of autophagy, is a crucial pathway for mitochondrial quality control where faulty mitochondria are sequestrated into autophagosomes for subsequent lysosomal degradation (Youle and Narendra, 2011; Kerr et al., 2017). Mitophagy dysfunction has been implicated in aging and multiple neurodegenerative diseases, such as AD, PD, ALS, and HD (Chu, 2019; Cai and Jeong, 2020). In this section, we offer a detailed and timely description of the molecular mechanisms of mitophagy and discuss current therapeutic approaches that target mitophagy and improve mitochondrial function in AD. In studies of yeast, worms (*Caenorhabditis elegans*), fruit flies (*Drosophila melanogaster*), zebrafish (*Danio rerio*), and mammals such as human (*Homo sapiens*), the molecular machinery that mediates the targeting of mitochondria to lysosomes has been elucidated (Lazarou et al., 2015). Importantly, PTEN-induced putative kinase protein 1 (PINK1)-parkin-mediated mitophagy is the most widely studied mitophagy pathway (Gautier et al., 2008). When mitochondria become damaged due to cellular stress, continued depolarization of their inner membrane occurs, leading to loss of mitochondrial ΔΨm, and this stabilizes PINK1 in the OMM. There, PINK1 phosphorylates Mfn2 and then stimulates the ubiquitin-proteasome system (UPS), which, in turn, recruits parkin to the OMM (Ziviani et al., 2010; Chan et al., 2011). This further promotes the engulfment of damaged mitochondria by the phagophore or isolation membranes and hence the formation of mitophagosomes destined for removal via the lysosomal system. Numerous studies have confirmed the roles of PINK1 and Parkin in mitochondrial quality control and mitophagy (Sung et al., 2016; Lee et al., 2018).

Mitochondrial quality control mechanisms that effectively sense and eradicate damaged mitochondria are weakened due to usage, aging, or disease, and this is likely to have a marked impact on neuronal health. A growing body of evidence indicates that inhibition of the clearance of damaged mitochondria and the concomitant increase in ROS results in an accumulation of impaired neurons in AD. One important aspect is that mitophagy could be compromised in AD due to unstable fusion of lysosomes and autophagosomes. Thus, disrupted lysosomal activity in healthy cells results in neuronal phenotypes resembling those in AD (Nixon et al., 2008). In addition, autophagosome aggregation develops after oxidative stress in mouse cortical neurons, which shows similarities with AD (Boland et al., 2008), while mutations in PS1 can impair autophagy/mitophagy (Lee et al., 2010). Together, these results indicate that impaired mitophagy is implicated in neuronal degeneration in AD (Figure 3).

## Strategies to Improve Mitochondrial Function in AD

The above analysis makes it clear that strategies capable of targeting mitochondrial function are needed to slow the progression of AD. The focus of the research effort should be to develop a therapeutic intervention that can target ROS and excessive mitochondrial fragmentation, thereby minimizing mitochondrial dysfunction and consequent synaptic injury during AD progression (summarized in Table 1). One fascinating approach to reducing the burden of ROS and improving mitochondrial health is exercise. In the following section, we discuss in detail the impact of physical activity on mitochondrial function.

**TABLE 1:** Summary of drugs aiming to improve mitochondrial dysfunction in AD.

Compounds	Mode of action	Therapeutic effects	References
Coenzyme CoQ10	Enhance electron transport chain	Mitigate ROS and enhance mitochondrial biogenesis	McCarthy et al., 2004; Singh et al., 2007; Sohet et al., 2009; Lee et al., 2013; Noh et al., 2013
Creatine	Buffer ATP	Mitigate ROS and enhance ATP	Matthews et al., 1998, 1999; Andres et al., 2008; Tarnopolsky, 2011
MitoQ	Enhancing electron transport chain	Mitigate ROS, enhance CREB signaling, improve mitochondrial health	Smith et al., 2003; Adlam et al., 2005; Cocheme et al., 2007; Xing et al., 2019
MitoVitE	Inhibit lipid peroxidation	Mitigate ROS, prevent apoptosis, inhibit cytochrome c and caspase-3 activity	Smith et al., 1999; Reddy, 2008; Jiang, 2014; Jameson et al., 2015
Sulforaphane	Nrf2 activation	Combat against ROS, upregulate cytoprotective genes, reduce inflammation, maintain redox homeostasis	Steele et al., 2013; Carrasco-Pozo et al., 2015
Bezafibrate	PGC1α activation	Increase biogenesis and ATP production	Chaturvedi and Beal, 2008; Steele et al., 2020
SS-31	Inhibit lipid peroxidation, PGC1α activation	Inhibit cytochrome c, reduce ROS, enhance ΔΨm	Manczak et al., 2010; Szeto and Birk, 2014; Reddy et al., 2018
Mdivi-1	Drp1 inhibitor	Decrease mitochondrial fission, reduce ROS, enhance biogenesis	Cassidy-Stone et al., 2008; Bido et al., 2017; Bordt et al., 2017; Smith and Gallo, 2017; Manczak et al., 2019
Dynasore	Drp1 and mTORC1 inhibitor	Inhibit mitochondrial fission, enhance biogenesis and mitophagy	Macia et al., 2006; Newton et al., 2006; Chung et al., 2010; Gao et al., 2013; Chen et al., 2019
DDQ	Inhibit Aβ and Drp1 binding	Decrease fission and increase fusion, increase PGC1α, Nrfl, Nrf2, and TFAM	Kuruva et al., 2017
P110	Drp1 inhibitor	Decrease fission and ROS, enhance ΔΨm, prevent apoptosis	Qi et al., 2013
SAMβA	Inhibit Mfn1 and βIIPKC binding	Increase fusion	Ferreira et al., 2019
BGP-15	Modulate OPA1 activity	Activate fusion	Szabo et al., 2018
Leflunomide	Modulate Mfnl/Mfn2 activity	Activate fusion	Miret-Casals et al., 2018
Ml	Modulate Mfnl/Mfn2 activity	Activate fusion	Wang et al., 2012; Peng et al., 2017
NAD^+^ precursors	Enhance NAD^+^ signaling	Enhance mitophagy, increase ROS resistance	Gong et al., 2013; Liu et al., 2013
DNP	Activate CREB, PGClα	Mitigate ROS, stimulate autophagy	Geisler et al., 2017
Rapamycin	mTOR inhibitor	Enhance mitophagy	Spilman et al., 2010
Urolithin A	Uncertain	Enhance mitophagy, reduce ROS	Ryu et al., 2016
Actinonin	Peptide deformylase inhibitor	Autophagy inducer	Richter et al., 2015
Spermidine	Decrease caspase-3 and p53	Enhance autophagy and cell survival, reduce ROS	Gupta et al., 2013; Jing et al., 2018

### Impact of Exercise and Diet on Mitochondrial Function and Oxidative Stress

Exercise is one of the most effective strategies for maintaining a healthy body and normal brain activity. Moreover, exercise and a healthy diet can specifically boost several aspects of mitochondrial function. In this context, the beneficial effects of caloric restriction and exercise in slowing the aging process and enhancing mitochondrial function have been shown in humans and rodent models (Barbieri et al., 2015; Lundby and Jacobs, 2016; Fiuza-Luces et al., 2019). Significantly, exercise not only enhances mitochondrial activity in the peripheral organs, but also completely blocks brain atrophy in mouse models. The beneficial effects of physical activity are now widely accepted in humans as a way to not only improve fitness, but also treat patients with neurodegenerative diseases, including AD (Bernardo et al., 2016). Additional studies have reported numerous advantages of exercise in AD patients, including better blood flow to the brain, enhanced hippocampal thickness, enhanced neurogenesis, cognitive performance, reduced neuropsychiatric symptoms, and slower disorder (Brown et al., 2013; Cass, 2017).

Worldwide, around 30% of adults are insufficiently active (Hallal et al., 2012), which is a greater risk of ROS-induced anomalies. It is well established that a sedentary lifestyle contributes to increased ROS and neuroinflammation seen in neurodegenerative disorders. On the other hand, physical exercise can mitigate inflammation and oxidative stress (Gleeson et al., 2011; Mazzola et al., 2011). This attenuation might be one of the mechanisms responsible for improving several clinical aspects, for instance, attenuating cellular aging (Puterman et al., 2010) and increasing insulin sensitivity (Gordon et al., 2014). Moreover, physical exercise prevented ROS and normalized its various components, including thiobarbituric acid reactive substances (TBA-RS), superoxide dismutase (SOD), catalase (CAT), and glutathione peroxidase (GPx) in rats (Mazzola et al., 2011). Lack of exercise leads to an overall reduction in mitochondrial ETC activity in healthy individuals. Simultaneously, endurance training can improve ETC activity, and resistance training can stimulate the integration of satellite cells into existing muscle fibers. Furthermore, exercise can stimulate mitochondrial proliferation through enhancing PGC-1α and AMPK signaling (Kang and Li Ji, 2012), and causes a reduction in the levels of systemic inflammation (McGee et al., 2003). This increase in PGC-1α and AMPK further promotes mitochondrial biogenesis.

Adequate consumption of vitamins and minerals and the use of natural foods rich in antioxidants (fruits, vegetables, etc.) could represent the ideal approach to maintaining the optimal antioxidant status. Foods that are rich in vitamin C can alleviate ROS. Vitamin C at various dosages, administered alone or in conjugation with other antioxidants, acutely or chronically, is the most commonly used antioxidant in clinical and laboratory research (Carr and Maggini, 2017; Spoelstra-de Man et al., 2018). Vitamin C attenuates ROS and maintains mitochondrial health in cells (Kc et al., 2005; Peng et al., 2019) and animal models (Singh and Rana, 2010). Similarly, Vitamin C consumption reduces Aβ plaque, preserves mitochondrial morphology, and ameliorates AD pathology in 5XFAD mice of AD (Kook et al., 2014). Moreover, the beneficial effect of caloric restriction on mitochondrial health is well documented. For example, a 15–25% continuous reduction in calorie intake in healthy adults for 24 months resulted in improved quality of life and a significant decrease in ROS levels (Redman et al., 2018). The caloric restriction mechanisms comprise activation of autophagy/mitophagy via AMPK-dependent inhibition of mTOR signaling, and the activation of Sirt1, which are important modulators of resistance to cellular stress, aging, and cell death. Moreover, caloric restriction has been shown to increase mitochondrial biogenesis and turnover, leading to a lesser accumulation of dysfunctional organelles, improved mitochondrial dynamics, morphology, and decreased mitochondrial permeability to Ca^2+^ retention capacity, ultimately leading to protection against excitotoxicity, a major mechanism involved in AD pathogenesis (Amigo et al., 2017). In addition to caloric restrictions, the ketogenic diet can slow down the development of cognitive dysfunction in patients with mild cognitive impairment (MCI) and AD. The neuroprotective effect of the ketogenic diet in AD is attributed to ketone bodies’ ability to provide a more efficient energy fuel source for mitochondria under conditions where glucose uptake is altered. Furthermore, ketone bodies have been shown to improve mitochondrial respiration, reduce the ROS production, improve antioxidant defense mechanism, and inhibit mPTP opening, thus ultimately protect mitochondrial and neuronal function (Masino and Rho, 2012). Overall, these studies suggest that physical exercise and diet have beneficial effects on mitochondrial health, redox homeostasis, and neuronal function, supporting the adoption of a healthy lifestyle as an invaluable tool against AD.

### Strategies to Mitigate Oxidative Stress and Enhance Mitochondrial Biogenesis

Antioxidant therapies, innovative pharmacological strategies designed to boost mitochondrial function, and mitigate local ROS production in mitochondria competing to reduce global levels of ROS. These compounds include coenzyme CoQ10, idebenone, creatine, MitoQ, MitoVitE, MitoTEMPOL, sulforaphane, bezafibrate, latrepirdine, methylene blue, triterpenoids, a series of Szeto-Schiller (SS) peptides such as SS-31, curcumin, Ginkgo biloba, omega-3 polyunsaturated fatty acids (Murphy and Hartley, 2018) and resveratrol which indirectly activates PGC-1α and induces mitochondrial biogenesis (Lagouge et al., 2006; Chuang et al., 2019). Numerous laboratories have extensively evaluated these mitochondrial-targeted compounds using *in vivo* and *in vitro* models of AD. Advantages of these compounds include improving bioenergetics, reducing ROS, maintaining mitochondrial dynamics.

#### Coenzyme CoQ10

CoQ_10_ is an essential cofactor of the ETC, functions by maintaining the mitochondrial ΔΨm, supporting ATP synthesis, and inhibiting ROS generation, thus protecting neuronal cells from oxidative stress and neurodegenerative diseases (McCarthy et al., 2004). Furthermore, it protects the membrane phospholipids, and mitochondrial membrane proteins against the damage of free radicals, increases mitochondrial mass and bioenergetic function (Singh et al., 2007; Noh et al., 2013). Other studies established that daily administration of CoQ10 significantly increased antioxidant enzyme activities and reduced inflammation (Sohet et al., 2009; Lee et al., 2013). Idebenone is an analog of CoQ10 that has better potency and a more promising pharmacokinetic profile. Idebenone can protect vision loss by enhancing mitochondrial ETC, in individuals with discordant visual acuities (Klopstock et al., 2011). CoQ10 has been utilized in many disease states to reduce ROS and improve mitochondrial health; this warrants CoQ10 use in preclinical and controlled human trials.

#### Creatine

Creatine in mitochondria combines with phosphate to form phosphocreatine, which functions as a source of high-energy phosphate released during anaerobic metabolism. Thus, creatine serves as an intracellular buffer for ATP and as an energy shuttle for the movement of high energy phosphates from mitochondrial sites of production to cytoplasmic sites of utilization. Creatine is present in the highest concentration in tissues with high energy demands, such as muscle and brain (Parikh et al., 2009). Studies suggest reduced phosphocreatine levels in muscle tissue were shown in individuals with mitochondrial dysfunction (Tarnopolsky and Parise, 1999), and administration with creatine monohydrate can enhance exercise capacity in some individuals with mitochondrial dysfunction (Tarnopolsky, 2011). Similarly, beneficial effects of creatine supplementation have been shown in neurodegenerative and neurological diseases linked with mitochondrial dysfunction, such as PD, HD, and ALS (Andres et al., 2008). Findings from rodent research suggest that creatine exerts neuroprotective effects by buffering ATP levels to counter neurotoxic assaults by mPTP opening, and malonate (Matthews et al., 1998, 1999). These data implicate that oral creatine may serve as a potential therapy against ROS and subsequent reduction of bioenergetics, which occurred in AD.

#### MitoQ

MitoQ is a mitochondria-targeted compound that enhances the mitochondrial protection against oxidative damage (Cocheme et al., 2007). MitoQ consists of a lipophilic cation moiety that enables mitochondria-specific accumulation and ubiquinone converted to the antioxidant ubiquinol by the activity of complex II of the ETC (Smith et al., 2003). MitoQ, water-soluble that can be administered orally through the drinking water, and can cross the blood–brain barrier (Rodriguez-Cuenca et al., 2010), have protective effects against mitochondrial alterations induced by oxidative stress in animal models (Adlam et al., 2005). Further, MitoQ prevented cognitive decline and neuropathology in a mouse model of AD (McManus et al., 2011). Treatment with MitoQ causes activation of cAMP response element-binding protein (CREB), thus improve mitochondrial health (Xing et al., 2019). Overall, these studies suggest the antioxidant and mitochondria protecting role of MitoQ in many pathological conditions, including AD.

#### MitoVitE

Vitamin E belongs to a group of compounds that includes both tocopherols and tocotrienols (Jiang, 2014). Tocopherol can protect cell membranes from oxidation, reacting with lipid radicals produced formed during lipid peroxidation (Jiang, 2014). MitoVitE is basically the chromanol moiety of vitamin E that bounds to a triphenyl phosphonium (TPP) cation and accumulates within mitochondria due to the large negative charge of the IMM. MitoVitE has been shown to accumulate in all major organs of mice and rats after oral, intraperitoneal, or intravenous administration and exerts a potent antioxidant activity (Jameson et al., 2015). Trolox, a synthetic, water-soluble, and cell-permeable derivative of vitamin E, often serves as a potent antioxidant in several model organisms (Wu et al., 1990; Guo et al., 2012). MitoVitE was more effective *in vitro* and *in vivo* than trolox (Jameson et al., 2015). MitoVitE can protect mitochondria from oxidative damage by reducing H_2_O_2_, inhibiting caspase activation, and blocking apoptosis (Reddy, 2008). Another study demonstrates that MitoVitE can prevent the release of cytochrome c, and staving off apoptosis by inhibiting caspase-3 activation, thus, rejuvenating ΔΨm for effective bioenergetics (Smith et al., 1999). Importantly, antioxidants which specifically accumulate within the mitochondrial matrix are suggested to offer better protection against oxidative stress.

#### Sulforaphane

Sulforaphane, a natural isothiocyanate-derived from a glucosinolate found in cruciferous vegetables, particularly broccoli, which is considered to be a common activator of Nrf2, can combat oxidative damage in mitochondria (Carrasco-Pozo et al., 2015). The activation of the Nrf2 pathway leads to upregulation of many downstream products involved in protection against oxidative stress, including NAD(P)H quinone oxidoreductase 1 (NQO1), heme oxygenase 1 (HO-1), glutathione peroxidase 1 (GPx1), and gamma-glutamylcysteine synthetase (γGCS) (Steele et al., 2013). Sulforaphane has powerful antioxidant and anti-inflammatory properties, which allow it to reduce cytotoxicity and ROS dramatically. Animal studies suggest that sulforaphane supplementation could be disease-modifying for many common, devastating neurological conditions, such as AD, PD, epilepsy, stroke, etc. Collectively, these results indicate that Nrf2 activators can have antioxidant effects by retaining mitochondrial redox homeostasis. Sulforaphane is a potential neuroprotective phytochemical that needs further human trials to determine its effectiveness in preventing and reducing the burden of multiple neurological diseases, including AD.

#### Bezafibrate

Through mitochondrial biogenesis, cells increase their mitochondrial population in response to increased energy demand. This is driven by the PGC-1α activation, which is a transcriptional coactivator that controls mitochondrial biogenesis. Bezafibrate, a peroxisome proliferator-activated receptor (PPAR) agonist widely used to treat dyslipidemia. Bezafibrate supplementation resulted in the initiation of mitochondrial biogenesis, leading to an increase in mitochondrial mass, oxidative phosphorylation capacity, and energy generation (Chaturvedi and Beal, 2008; Steele et al., 2020). These findings imply that bezafibrate might be a promising therapeutic agent for treating any neurodegenerative diseases associated with mitochondrial dysfunction.

#### SS-31

SS-31 is a small molecule that has been shown to exert potent antioxidant effects against ROS to protect mitochondrial function. SS-31 can prevent the peroxidase activity of cytochrome c in mitochondria, reduce ROS production, and aid reversal of mitochondrial dysfunction (Szeto and Birk, 2014). Moreover, SS-31 was proven to inhibit lipid peroxidation and hydrogen peroxide scavenging (Reddy et al., 2018). SS-31 has the benefit of being localized to the mitochondrion, explicitly targeting the IMM rather than the mitochondrial matrix. Treatment with SS-31 prevented mitochondrial dysfunction and enhanced ΔΨm, and increased neuroprotective gene PGC-1α in neuroblastoma N2a cells grown with mutant APP (Manczak et al., 2010), suggesting beneficial effects of the drug on mitochondrial alterations in AD. It is worth noting that mitochondria-targeted small molecules such as SS-31, have been tested in cell culture and animal models of neurodegenerative diseases, such as AD, HD, PD, ALS, multiple sclerosis, and other human diseases. Therefore, it is important to consider using small molecules for preclinical models and human clinical trials.

### Strategies to Inhibit Excessive Mitochondrial Fission in AD

#### Mitochondrial Division Inhibitor 1 as a Mitochondrial Fission Inhibitor

In the last two decades, some inhibitors of Drp1, including, in particular, mitochondrial division inhibitor 1 (Mdivi-1), dynasore, diethyl (3,4-dihydroxyphenethylamino) (quinolin-4-yl) methylphosphonate (DDQ), and P110, have been developed, and their beneficial effects have been studied in cell cultures and mouse models. The quinazolinone derivative, Mdivi-1, identified initially as a selective inhibitor of mitochondrial fission protein DRP1, induces neuroprotection in AD and PD models, as well as other neurodegenerative disorders, by improving mitochondrial fusion, and increasing mitochondrial biogenesis and synaptic protein levels (Bido et al., 2017; Smith and Gallo, 2017; Manczak et al., 2019). Recently, Mdivi-1 was shown to serve as a reversible mitochondrial complex I inhibitor that decreases mitochondrial fission and ROS production and further enhances mitochondrial function (Bordt et al., 2017). In addition, recent detailed studies of neuronal N2a cells support the theory that Mdivi-1 inhibits mitochondrial heterogeneity and increases energy efficiency (Manczak et al., 2019). On the other hand, the capacity of Mdivi-1 to suppress Drp1 and trigger mitochondrial fission has recently been questioned (Bordt et al., 2017). The authors did not notice any treatment effect with Mdivi-1 on mitochondrial morphology in mammalian cells in this study. However, they confirmed the impact of Mdivi-1 on yeast Drp1, consistent with a previous report (Cassidy-Stone et al., 2008). As Mdivi-1 is being considered for clinical trials, it may be appropriate to carry out more thorough investigations into its molecular targets to ensure its safety and effectiveness in humans.

#### Dynasore as a Mitochondrial Fission Inhibitor

Another cell-permeable small molecule that inhibits Drp1 activity is dynasore (Macia et al., 2006). A low dose of dynasore is sufficient to inhibit mitochondrial fission caused by ROS in cultured cells (Gao et al., 2013), which, in turn, inhibits endocytosis in neuronal cells and phagocytosis (Newton et al., 2006; Chung et al., 2010). Furthermore, dynasore inhibits mTORC1, which leads to nuclear translocation of TFEB and TFE3, the master regulators of autophagy and lysosomal biogenesis, thereby increasing autophagic flux. Dynasore therapy greatly improves the clearance by autophagy of protein aggregates of mutant HD in cells (Chen et al., 2019). In this context, a recent study suggests dynasore treatment decreases Aβ internalization and processing to the secretory pathway. However, there is currently a lack of data on the impact of dynasore treatment on fission-fusion and mitophagy in early and late-onset AD. Nevertheless, pharmacological interventions that inhibit the actions of Drp1 provide hope that excessive mitochondrial fragmentation can be abrogated in AD. Finally, the inhibition of the fission mechanism will benefit mitophagy, and energy production.

#### DDQ as a Mitochondrial Fission Inhibitor

Recently, the role of mitochondrial dysfunction in AD has been studied using the pharmacological compound diethyl (3,4 dihydroxyphenethylamino) (quinolin-4-yl) methylphosphonate (DDQ). DDQ has shown promising effects on mRNA and protein levels associated with mitochondrial dysfunction and AD-related synaptic dysregulation. In addition, DDQ decreases mitochondrial fission proteins (Drp1 and Fis1), increases fusion proteins (Mfn1 and 2), and inhibit Aβ interactions. A novel property of DDQ is that it binds at the active binding sites of Aβ and Drp1, inhibiting the formation of complexes between of Aβ and Drp1 (Kuruva et al., 2017). This research indicates that DDQ can decrease the levels of Drp1 and Aβ, inhibit irregular Drp1-Aβ interactions, further decrease excessive mitochondrial fragmentation, and maintain mitochondrial function and synaptic activity in AD neurons. The effect of DDQ either before or after Aβ treatment on levels of various mRNAs and proteins important for mitochondrial function (PGC-1α, Nrf1, Nrf2, TFAM, DRP1, Fis1, Mfn1, and Mfn2), as well as those involved in synaptic activation (synaptophysin, PSD95, synapsin1 and 2, synaptobrevin1 and 2, synaptopodin, and GAP43), has also been examined. The mRNA and protein levels of mitochondrial-enhancing molecules such as PGC-1α, Nrf1, Nrf2, and TFAM were substantially increased following incubation with Aβ, followed by DDQ treatment. In addition, after DDQ therapy, a decrease in levels of mitochondrial fission proteins (DRP1 and Fis1) and an increase in levels of mitochondrial fusion proteins (Mfn1 and 2) was observed. This led to the conclusion that in the presence of Aβ, pretreatment with DDQ decreases fission activity (DRP1 and Fis1) and increases fusion activity (Mfn1 and 2). In order to evaluate its protective effects against Aβ-induced neuronal toxicity, more preclinical research using AD mouse models and clinical trials using AD patients treated with DDQ are needed.

#### P110 as a Mitochondrial Fission Inhibitor

P110 is another inhibitor of Drp1, which acts by blocking the interaction of Drp1 and Fis1. P110 was first used to protect neuronal cells: in cultured neurons, it reduces mitochondrial fragmentation and generation of ROS, restores mitochondrial integrity and ΔΨm, and protects cells against apoptosis caused by ROS (Qi et al., 2013). Other studies have used P110 to inhibit mitochondrial fission, thereby protecting the cell from death caused by stress or damage, especially in heart disease models. Moreover, treatment with P110 increases acute infarction-induced cell death and reduces heart failure both *in vitro* and *in vivo* (Disatnik et al., 2013). Therefore, P110 treatment should be assessed in early- and late-onset AD, at least in animal models, to confirm the therapeutic effects of this compound on mitochondrial fragmentation.

### Strategies to Enhance Mitochondrial Fusion

Therapeutic interventions directed at the fusion machinery (Mfn1, Mfn2, and OPA1) will enhance mitochondrial health by optimizing and rebalancing the regulation and control of fusion. Drugs that improve mitochondrial fusion function have recently been documented to suppress the death of apoptotic cardiac cells *in vivo*. When the association between Mfn1 and βIIPKC is blocked by the novel agent SAMβA, mitochondrial fusion and cardiac function are enhanced in rats (Ferreira et al., 2019). Another promising compound, which modulates the activity of OPA1 in lung epithelial cells, is the small molecule BGP-15 (Szabo et al., 2018). Leflunomide, a new compound launched in 2018, is another small molecule that activates fusion, as recognized by Mfn1/Mfn2-dependent mitochondrial elongation. Leflunomide-treated HeLa cells display an elongated mitochondrial network and enhanced expression of Mfn1/2. Mechanistically, leflunomide inhibits pyrimidine synthesis; this minimizes the activity of doxorubicin-induced PARP and cleaved caspase in3 in embryonic mouse fibroblast (MEF) cells and shields PC12 cells from apoptosis (Miret-Casals et al., 2018). In 2012, another mitochondrial fusion activator hydrazone M1 was introduced. Mitochondrial fragmentation is common in SH-SY5Y cells treated with 1-methyl-4-phenyl-pyridinium-(MPP+-), which models the death of neurons in PD. Treatment with the fusion promoter M1 in this model reduces the release of cytochrome c and prevents cell death (Wang et al., 2012). Similarly, M1 also protects mitochondrial function in a rotenone-induced PD model (Peng et al., 2017). These findings indicate that maintaining mitochondrial fusion is a promising approach to the treatment of a number of human diseases, including AD. Thus, further research is warranted, at least in animal models of AD, to test the effects of these drugs on impaired fusion machinery in AD.

### Strategies to Enhance Mitophagy

Pharmacological agents and lifestyle interventions targeted at enhancing mitophagy are a promising approach for achieving a significant therapeutic benefit (Kerr et al., 2017; Lou et al., 2020). Caloric restriction, prolonged fasting, and physical exercise are bioenergetic challenges that can enhance neuroplasticity (i.e., they promote synapse formation and hippocampal neurogenesis, reduce ROS, stimulate mitochondrial biogenesis, and enhance autophagy) (Longo and Mattson, 2014). For example, in mice, fasting and exercise result in elevated numbers of autophagosomes in cerebral cortical neurons, increased expression of SIRT3, and activation of mitochondrial biogenesis by a pathway involving BDNF signaling and PGC-1α upregulation (Alirezaei et al., 2010; Cheng et al., 2012, 2016). Thus, exercise and fasting can augment the numbers of healthy mitochondria in neurons by promoting the pathways that enhance mitophagy. A variety of mitophagy-enhancing compounds in AD and other neurodegenerative diseases have been investigated recently, including nicotinamide adenine dinucleotide (NAD^+)^ precursors, urolithin A (Ryu et al., 2016), the antibiotic actinonin (Richter et al., 2015), and spermidine (Gupta et al., 2013). NAD^+^ levels are reduced in animal models of AD, and elevation of cellular NAD^+^ levels by treatment with NAD^+^ precursors such as nicotinamide, nicotinamide mononucleotide, and nicotinamide riboside attenuates Aβ and tau pathologies, improves SIRT3 function, increases mitochondrial resistance to ROS, enhances mitophagy and prevents cognitive dysfunction, likely by upregulating the activity of the CREB transcription factor (Gong et al., 2013; Liu et al., 2013). In addition, mitochondrial uncoupling agents, such as 2,4-dinitrophenol (DNP), can induce autophagy and are useful in maintaining neuronal activity in animal AD models (Geisler et al., 2017). The mTOR inhibitor, rapamycin, is another mitophagy-inducing drug that can prevent cognitive defects and reduce Aβ pathology in an APP-mutant AD mouse model (Spilman et al., 2010). Collectively, these results provide encouragement for controlled human trials to explore the therapeutic potential of the above compounds.

**FIGURE 4:**
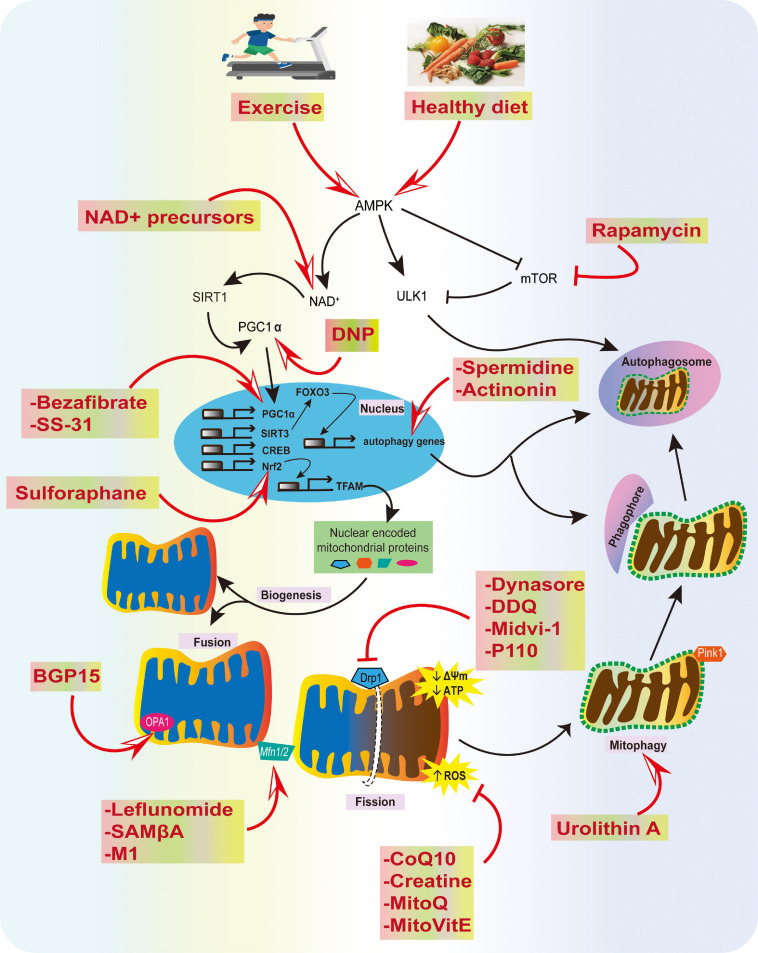
Summary of physiological and pharmacological interventions and molecular targets to improve mitochondrial function in AD. We highlight several strategies that can contribute to mitochondrial health, such as exercise and a healthy diet, inhibition of excessive mitochondrial fragmentation and ROS, and improving fusion, biogenesis, transport, and mitophagy using various compounds, as potential strategies for AD prevention.

One approach that might accelerate the translation of these mitochondrial agents into the clinic is to screen compounds in animal models at the prodromal stage and after neurological symptoms. If the drug impedes disease development in these models, there would be a firm basis for moving to human trials. We remain enthusiastic about the prospects for the treatment of neurodegenerative diseases using mitochondrial therapies; specifically, those designed to prevent mitochondrial damage, stimulate organelle biogenesis, and improve mitochondrial quality control (Figure 4). However, these advances require improvements in early diagnosis, the development of clinically appropriate biomarkers, and better trial design to allow for more rapid identification of compounds for the clinic.

### Challenges in Mitochondria-Targeted Strategies

Apart from offering a cure, most therapeutic interventions can have numerous adverse side effects, which can also be the case with the use of some mitochondria-targeted therapeutics. Targetting mitochondria is a novel strategy; nevertheless, despite very encouraging results in using these mitochondrial-targeted therapeutics, it is challenging to target the population of damaged mitochondria selectively. Notably, the targeting of compounds to the subcellular compartments represents one of the modern molecular pharmacology trends. The drug substance’s target is located inside an intracellular compartment such as a mitochondrion, and the drug molecule has to penetrate several membranes to reach the final destination. The molecule would require exact physicochemical properties to cross the different barriers. For example, to suppress ROS, antioxidants administered orally or intraventricularly, or intra-muscularly, must travel through the blood and finally reach the targeted organ. However, in this case, healthy tissues other than targeted organs that have not undergone oxidative damage, could be unavoidably targeted by frequent use of these antioxidants. As a result, upon increasing antioxidant doses to permit the repair of pathological mitochondria (especially toxic hyperpolarized mitochondria), normal mitochondria may also be adversely affected, as their ROS levels may fall below their physiologically acceptable limit. Undoubtedly, these compounds protected mitochondrial health and delayed aging in various animal trials; however, clinical evidence has not fully supported these preliminary findings. Thus, we have to be mindful of dosage, timing, and modes of exposure for different pathologies, with all the apparent benefits of mitochondrial-targeted therapeutics.

## Future Perspectives

Significant progress has been made in discovering the causes of the debilitating neurodegenerative disorder AD. Accumulating data suggest that mitochondria play a vital role in the pathogenesis of AD by causing increased ROS production and oxidative damage, disturbance of Ca^2+^ homeostasis, activation of the mPTP, and alterations in dynamics, and mitophagy. The exploration of treatments that target mitochondria in AD is in progress, but there is an immediate need to develop novel therapeutic approaches that block or slow down the progression of this incurable disease. Here, we reviewed novel strategies for targeting altered pathways based on an aggregation of misfolded protein, defects in mitochondrial dynamics, OXPHOS dysfunction, oxidative stress, and compromised mitophagy in AD pathology. It remains a significant challenge to develop mitochondrially targeted AD therapeutics using innovative drug delivery systems and transfer them from the lab bench to the hospital bed. Nevertheless, in our opinion, focusing on mitochondria and expanding the area of mitochondrial pharmacology has enormous potential for modern, reliable mitochondrial therapy in AD.

## Author Contributions

AM, and LY designed the theme of the manuscript. AM contributed writing the following sections “Introduction,” “Oxidative Stress and Mitochondrial Defects in AD,” “Impaired Mitochondrial Dynamics and Mitophagy in AD,” “Strategies to Improve Mitochondrial Function in AD,” and created Figures 1, 4. ST wrote “Shortage of Neuronal ATP in AD” and “Mitophagy Defects in AD” and made Figures 2, 3. LY conducted a critical revision of the manuscript. All authors contributed to the article and approved the submitted version.

## Conflict of Interest

The authors declare that the research was conducted in the absence of any commercial or financial relationships that could be construed as a potential conflict of interest.
